# Rituximab in relapsing and de novo MPO ANCA-associated vasculitis with severe renal involvement: a case series

**DOI:** 10.1186/s12882-019-1350-x

**Published:** 2019-05-14

**Authors:** L. Caroti, C. L. Cirami, L. Di Maria, A. Larti, P. Carta, E. Dervishi, S. Farsetti, A. Tsalouchos, L. Novelli, E. E. Minetti

**Affiliations:** 1 Nephrology Unit, Careggi University Hospital, Florence, Italy; 2 Department of Medical and Surgical Critical Care, Division of Pathological Anatomy, University of Florence, Florence, Italy

**Keywords:** MPO-ANCA associated vasculitis, Severe renal involvement, Rituximab

## Abstract

**Background:**

Antineutrophil cytoplasmic antibody associated vasculitis (AAV) is a group of diseases associated in most cases with the presence of anti-neutrophil cytoplasmic antibodies (ANCAs). Rituximab- based remission induction has been proven effective in ANCA associated vasculitis but scarce data exist in forms with severe renal involvement. In this case series, we report the outcomes in patients with de novo or recurrent MPO-AAV and severe renal involvement treated with rituximab without cyclophosphamide (CYC).

**Methods:**

In this single centre retrospective study, we analysed patients with a clinical diagnosis of de novo or recurrent AAV who met the following criteria: detection of P-ANCA, creatinine clearance lower than 30 ml/min, induction of remission therapy with rituximab without concomitant CYC and a follow up period of at least 6 months. The primary outcomes were complete remission after induction therapy, renal function recovery and mortality after the induction treatment.

**Results:**

Eight patients met the inclusion criteria. The M:F ratio was 1:7, the average age was 54 years old and the median follow up was 10 months (7–72); in 2 patients there was a MPA renal limited vasculitis. A renal biopsy was performed in 7 patients. The median BVAS score at rituximab induction was 14(range 6–21). Two patients required haemodialysis before the induction treatment. Four patients developed end stage renal disease (ESRD) that required haemodialysis. These data show a remission of the disease, associated with a stabilization of the kidney function in 50% of patients. In 3 patients who did not show a response, there was also no response to CYC.

**Conclusions:**

This study shows a partial efficacy of rituximab in renal function recovery and a low risk of infectious complications in patients with MPO vasculitis with severe renal involvement, in particular in the short term. The optimal treatment in this subgroup of patients still has to be established because data are lacking.

## Background

Antineutrophil cytoplasmic antibody associated vasculitis (AAV) is a group of necrotizing pauci-immune multi systemic diseases characterised by glomerulonephritis with absence or paucity of immunoglobulin deposits. The most common forms of AAV are granulomatosis with polyangiitis (GPA), microscopic polyangiitis (MPA) and eosinophilic granulomatosis with polyangiitis (EGPA) [1–4]. In most cases, AAV is associated with the presence of anti-neutrophil cytoplasmic antibodies (ANCAs). Many studies have documented that the most important factors associated with a bad prognosis of AAV are age at onset, markedly decreased glomerular filtration rate (eGFR< 15 ml/min), a high Birmingham Vasculitis Activity Score (BVAS), pulmonary involvement at presentation and high levels of PR3 ANCA [5]. Renal involvement at onset is a major predictor for the outcome in AAV; patients with PR3-AAV have a greater risk of relapse while MPO ANCA patients are characterised by a worse renal function and higher levels of proteinuria at the onset of the disease [6]. Treatment of AAV is considerably improved since the introduction of immunosuppressive therapy that consist of an induction of the remission phase (3–6 months), followed by a remission maintenance (for at least 18 months), associated with plasma exchange in patients with a severe or life-threatening form of the disease. The introduction of corticosteroids and cyclophosphamide (CYC) as an induction therapy in AAV improved patients’ survival from 20% to over 80% at 2 years [7–9]; therefore CYC is the treatment of choice for the induction of remission in AAV. However treatment with CYC is associated with a high risk of infectious complications, cardiovascular disease, myelosuppression and malignancy [10, 11]. Infertility is another major problem, however there are anecdotic cases of successful conceptions and pregnancies in p-ANCA associated vasculitis during CYC treatment [12]. Taking into consideration the importance of a prolonged therapy with CYC and the high relapse rate of the disease, many therapeutic trials showed less severe adverse events and no less efficacy using less toxic induction treatment or reduced dose of intravenous pulse CYC versus standard dose [5]; however CYC sparing strategies seem to show a higher risk of relapse. Many other studies have been performed to show less toxicity with alternative treatments for remission induction; most of them are limited by a small cohort of patients with severe renal involvement [13]. Rituximab (RTX) has been used in ANCA associated vasculitis since 2003. The RAVE [14] and RITUXVAS trials [15] showed no difference between the anti-CD20 antibody RTX versus CYC for remission induction in patients with GPA and MPA. Unfortunately the RAVE trial evaluated new or relapsing GPA or MPA in patients without severe renal involvement and in the RITUXVAS trial all patients with severe renal disease also received CYC in the induction phase. Thus, there are very few data about the outcome in patients with severe renal involvement treated only with RTX and steroids for remission induction. In a recent multi-centre retrospective study [16], Shah S et al. reported a favourable outcome in terms of remission and dialysis independence in patients with severe renal involvement treated with RTX. In many clinical trials both MPA and GPA are included; however it is debatable if they represent distinct diseases or a continuum, and current classification systems sometimes confuse the distinction of these two clinical entities. Moreover some authors suggest a distinction in the ANCA type, not just based on clinical features, for the characterisation of the disease. In this single centre retrospective study, we report the clinical outcomes in 8 patients with de novo or recurrent MPO-ANCA associated vasculitis and severe renal involvement treated with RTX with no CYC association. We treated this group of patients with RTX without CYC taking into account the presence of comorbidities and the high risk of toxicity and infectious complications in this specific population. Furthermore, there is growing evidence about the efficacy of RTX in patients with severe renal involvement [16, 17, 18]. We have focused this study on the subgroup of MPO vasculitis because MPO are more often associated with crescentic glomerulonephritis, renal involvement [17], failure to obtain complete remission and poorer renal outcome. In addition, post-hoc analysis in the RAVE trial showed that RTX was more effective in remission induction in the PR3 ANCA group than in the MPO group.

## Methods

### Study population

In this monocentric retrospective study, we identified patients from medical records and an electronic database between 2001 and 2017. Patients who met the following criteria were included: 1) detection by indirect immunofluorescence and ELISA of anti-neutrophil cytoplasmic antibody P-ANCA; 2) creatinine clearance (mGFR) less than 30 ml/min; 3) induction of remission therapy with RTX without concomitant CYC; 4) follow up period of at least 6 months; 5) clinical diagnosis of de novo or recurrent rapidly progressive glomerulonephritis. Patients may have had plasma exchange and glucocorticoids. The study protocol was approved by the institutional review board of our institution.

### Clinical and laboratory data

All clinical and laboratory data were recorded at the time of inclusion taking into consideration clinical source documents. All the patients included in the study were evaluated and the following data reported: age at induction, gender, kidney biopsy classified to International Working Group of Renal Pathologists (IWGRP), proteinuria, disease severity at diagnosis or relapse using version 3 of the BVAS, degree of renal involvement and the need of renal replacement therapy at diagnosis (RRT), type of induction therapy including the possible treatment with plasma exchange or high dose immunoglobulin, any complication during induction of remission therapy, maintenance therapy, VDI score (vasculitis damage index) at the end of follow up. Laboratory data were taken from an electronic laboratory database; we evaluated ANCA specificity using indirect immunofluorescence and ELISA. Renal involvement was based on clinical data and renal biopsy (excluding cases with contraindications). Renal function in the follow up was assessed using creatinine clearance or e-GFR (Table 1-2).

**Table 1:** Demographics data at the induction treatment with rituximab

Patient	Gender	Age	Vasculitis type	Relapse/De novo/	Kidney biopsy (IWGRP)^a^	CrCl^b^ (ml/min)	BVAS^c^	PE	HD	Time between CYC and RTX (months)
1	x	d	MPA Renal limited	Relapse	Crescentic	24	8	NO	NO	66
2	x	c	GPA	Relapse	Sclerotic	25	13	NO	NO	60
3	x	d	MPA Renal limited	Relapse	Crescentic	15	6	NO	NO	4
4	x	b	MPA	Relapse	Mixed	13	14	YES	NO	8
5	x	b	MPA	De novo	NA^d^	4	16	YES	YES	NA^d^
6	x	a	MPA	De novo	NA^d^	< 5	21	NO	YES	NA^d^
7	y	c	MPA	De novo	Mixed	22	21	NO	NO	NA^d^
8	x	d	MPA	De novo	Crescentic	11	12	NO	NO	NA^d^

^a^IWGRP: International Working Group of Renal Pathologists

^b^Creatinine clearance (ml/min)

^c^Birmingham Vasculitis Activity Score (version 3)

^d^Not applicable

Age: a (18–29), b (30–45), c (55–64), d (> 70)

**Table 2:** Outcomes of patients treated with rituximab

Patient	Rituximab scheme	CrCl^a^	Follow up (months)	Leukopenia	Infection	Dialysis	Rituximab maintainance therapy	VDI score^b^
1	375 mg/m^2^ weeklyx4	27	22	No	no	no	yes	4
2	1000 mgx2	NA^c^	72	No	no	yes	no	2
3	375 mg/m^2^ weeklyx4	21	7	No	yes	no	no	5
4	1000 mgx2	NA^c^	8	Yes	no	yes	no	NA^c^
5	375 mg/m^2^ weeklyx4	NA^c^	19	No	yes	yes	no	1
6	375 mg/m^2^ weeklyx4	NA^c^	11	Yes	yes	yes	yes	1
7	375 mg/m^2^ weeklyx4	95	9	No	no	no	yes	2
8	375 mg/m^2^ weeklyx4	20	12	No	no	no	yes	3

^a^Creatinine clearance (ml/min) or eGFR ml/min/1.73m^2^

^b^Vasculitis damage index

^c^Not applicable

### Outcomes

The primary outcomes that we assessed were complete remission after induction therapy with RTX after at least 3 months of treatment, renal function recovery, ESRD (defined as the need of renal replacement therapy after 6 months of treatment) and mortality after the induction treatment. Secondary outcomes were drug related clinical complications, onset of infections and cardiovascular disease (Table 2). Disease phenotype was fixed using the Chapel Hill Consensus nomenclature. Remission was defined using version 3 of the BVAS. At 6 months, patients with stable renal function without extrarenal disease activity were classified to have a BVAS score of 0. The VDI index was used to evaluate the degree of damage at the end of follow up.

## Results

Between 2001 and 2017 we diagnosed 35 cases of ANCA associated vasculitis; 11 of them received Rituximab alone as induction treatment without CYC. Patients with eGFR greater than 30 ml/min or affected by PR3 vasculitis were excluded. Eight patients met the inclusion criteria. The M:F ratio was 1:7, the average age was 54 years old and the median follow up was 10 months (7–72); in 2 patients there was a MPA renal limited vasculitis. In 7 patients we performed a renal biopsy: three of these were classified as crescentic class and 2 patients were classified as mixed class by the IWGRP system; in 1 case the kidney biopsy showed a sclerotic class. One patient underwent a kidney biopsy, but the specimen was not adequate for classification according to the IWGRP system. One patient did not undergo a kidney biopsy due to a clinical contraindication. The median BVAS score at Rituximab induction was 14 (range 6–21). Two patients required hemodialysis before the induction treatment. Four patients had a recurrent disease at the induction with Rituximab, and 4 patients had a newly diagnosed disease (two of them requiring hemodialysis at the onset). Two patients received plasma exchange (PE) during the induction of remission phase (Table 2); the other patients did not receive PE due to a clinical contraindication. The 4 patients treated with rituximab for a recurrence of the disease, received CYC as the induction treatment at the disease onset. The median time between the last dose of CYC and RTX was 33 months (range 4–66). Only one patient received CYC at a median time less than 6 months (Table 1), however the treatment was stopped after less than 2 months due to severe sepsis and pancytopenia; in this specific case we did not consider a complete induction treatment and RTX was administered due to failure in improvement in kidney function. All patients with systemic MPA or GPA achieved the remission after the induction treatment with RTX (Table 1). Two patients with MPA renal limited received RTX for a relapsing disease after an efficacious induction treatment with CYC or due to treatment related complications. In 4 patients clinical data showed a chronic persistent disease characterised by end stage renal disease requiring haemodialysis after 6 months of treatment. One of them received Rituximab for a relapsing disease after induction treatment with CYC and steroids (kidney biopsy showed a sclerotic class); one of them showed a refractory disease for CYC and RTX (Mixed class in IWGRP). The other 2 received RTX for a de novo ANCA associated vasculitis requiring haemodialysis at the onset (IWGRP class not available) showing no renal function recovery. Two patients showed a de novo disease treated with RTX achieving the remission of the disease and significant improvement of mGFR, one of them showed a severe fibrotic pulmonary involvement; two patients maintained independent renal function without significant recovery of renal function (Table 2). The induction scheme was RTX 375 mg/m^2^ weekly for 4 weeks in 6 cases or two doses of 1000 mg/eow in 2 cases; all patients also received methylprednisolone 15 mg/kg/die for 3 days followed by 1 mg/kg/die of prednisone for 1 months. Two patients received 3–5 sessions of plasma-exchange. One patients received PE after 3 doses of RTX. Four patients with remission after the induction received RTX as a maintenance therapy at the dose of 500 mg every 6 months. In all cases induction was well tolerated without severe complications. One patient developed pneumonia at the time of the third infusion treated successfully with antibiotic therapy.

## Discussion

Introduction of RTX in treatment of ANCA associated vasculitis was an important improvement in the management of the disease. Apart from the clinical spectrum of the disease, many patients also suffered from toxic effects due to high dose of CYC. The RITUXVAS and RAVE trials opened the way for use of RTX in AAV showing no difference in remission induction, leaving some questions in patients with severe renal involvement. In the 2012 KDIGO guidelines authors recommended CYC and corticosteroids as an initial treatment in ANCA associated vasculitis, reserving the use of RTX in patients without severe disease or when CYC is contraindicated or in relapsing/resistant disease [19]. Despite these indications in the last few years some authors reported the safety and efficacy of RTX without CYC in remission induction even in ANCA associated vasculitis with severe renal involvement. Recently the efficacy of RTX in patients with renal involvement has been reported (mean eGFR 41 ml/min) and it showed no difference when compared to CYC considering relapses and improvement in eGFR at 18 months [17]. Some authors have reported the outcome in 14 patients with very severe renal impairment (eGFR< 20 ml/min/1.73 m2) due to AAV or ANCA negative vasculitis [16]; this multicentre study showed 100% achieving remission and an improvement in kidney function in most of cases (both MPO and PR3 vasculitis). In this retrospective single centre study we have focused the analysis only in the subgroup of MPO vasculitis with severe renal involvement, because of scarce data in the use of RTX in these patients and the likely increased effectiveness of RTX in PR3 disease, as reported by some authors [20]. Moreover, data on RTX efficacy in patients with severe kidney disease are still insufficient. Taking into consideration these data, unlike previous studies, we have only considered this specific subgroup of patients. These data show a remission of the disease associated with an improvement of the kidney function in 2 patients and only an insignificant renal function recovery in 2 patients. Three of them had a crescentic vasculitis, 1 had a mixed form. In the 4 cases without a recovery of the kidney function, a kidney biopsy was suitable for 2 cases showing a sclerotic class in one and a mixed class in the other. These data confirm, as previously demonstrated by many authors, that a kidney biopsy is an important tool for the prediction of the response to the treatment. However a detailed analysis about the RTX impact on the recovery of renal function in this series remains difficult because it depends on several factors such as renal function at the time of diagnosis, chronicity score systems regarding histopathologic parameters (tubular atrophy, interstitial fibrosis, percentage of normal glomeruli and arteriosclerosis), presence of co-morbidities and ANCA type [21]. Moreover histopathologic scoring systems could predict the probability to develop ESRD regardless of immunosuppressive treatment [22]. The modest improvement in renal function apart from 2 patients could also be explained by the short follow up, therefore the complete recovery of renal function may be underestimated. We should also point out that in 1 patient who did not respond to RTX, we performed PE and intravenous CYC after 6 months without recovery of the renal function showing a refractory disease. These data suggest that probably, some of the cases where patients who did not show a remission induction with RTX, may also be resistant to CYC. An important factor that strongly plays in favour of RTX is the low risk of serious side effects, especially in the short term. In all patients we performed the VDI score at the end of the follow up for the clinical assessment of organ damage related to the disease and our data showed a very low value particularly in patients treated with RTX in de novo vasculitis with high BVAS at the onset (Table 2), in contrast with previous reports of higher VDI in the same setting as well as in relapsing vasculitis [23]. In all the cases rituximab was well tolerated, without reactions or clinical symptoms during the infusion. Four patients developed infection (pneumonia, enteritis) or leukopenia after the infusion without severe complications. Patients treated with rituximab for the induction who continued the treatment in the maintenance phase, showed no complications. However, it should be noted that the short follow up of this series could have underestimated the incidence of infectious complications taking into consideration the prolonged effect of Rituximab on B-cell depletion. It is well known that AAV patients present a high risk of developing serious infections and Rituximab has been associated with severe complications such as late-onset neutropenia and hypoimmunoglobulinaemia. Taking into considerations all these factors the importance of the assessment of the clinical risk factors and BVAS score before the induction treatment should be pointed out, in order to perform an individualized evaluation of the risk of infection. In this setting a particular attention should be paid to the development of *Pneumocystis jiroveci* pneumonia; some data suggest the prophylaxis with cotrimoxazole in all patients undergoing CYC or RTX treatment [24]. This study is limited by its retrospective design and by the small number of cases and short follow up, nevertheless it focuses on a homogeneous subgroup of patients all affected by MPO-AAV and severe renal involvement.

## Conclusion

This study confirms the efficacy of rituximab in remission induction in patients with MPO vasculitis with severe renal involvement and also its safety, at least in the short term, in these patients who are at particular risk of developing serious adverse effects with traditional, more potent, immunosuppressive approaches. It is more difficult to draw a conclusion regarding renal function recovery because of the limits of our study (small size, short term follow up and heterogeneity of our cohort) and because it can be influenced negatively by several factors such as histological predictors of ESRD. The optimal therapeutic approach in this subgroup of patients remains to be established. The lack of efficacy in renal function recovery seems to be in some cases limited to patients already known as non responders, however the effect of RTX seems not to be dependent on a previous response to CYC. Taking into considerations these data, we need a randomized multicentre study, with kidney biopsy as part of the initial assessment, in MPO-AAV patients with severe renal involvement. As recently discussed, the combined use of a low dose of CYC and Rituximab could be a valid option in MPO-AAV with severe renal involvement allowing a rapid response, prevention of long- term damage aiming to minimize steroid exposure [25].

## Abbreviations

AAV: Antineutrophil cytoplasmic antibody associated vasculitis
BVAS: Birmingham vasculitis activity score
CYC: Cyclophosphamide
EGPA: Eosinophilic granulomatosis with polyangiitis
ESRD: End stage renal disease
GPA: Granulomatosis with polyangiitis
IWGRP: International working group of renal pathologists
mGFR: measured glomerular filtration rate
MPA: Microscopic polyangiitis
VDI: Vasculitis damage index
